# The relationship between health belief and sleep quality of Chinese college students: The mediating role of physical activity and moderating effect of mobile phone addiction

**DOI:** 10.3389/fpubh.2023.1108911

**Published:** 2023-04-13

**Authors:** Xinchao Gao, Chuang Li, Beining Han, Peng Xu, Chenxu Qu

**Affiliations:** ^1^Physical Education Department, Northeastern University, Shenyang, China; ^2^Physical Education Department, Yuncheng Vocational and Technical University, Yuncheng, China; ^3^Department of Basic Education, Henan Polytechnic, Zhengzhou, China; ^4^School of Physical Education and Sport, Henan University, Kaifeng, China; ^5^School of Physical Education, Central China Normal University, Wuhan, China; ^6^School of Physical Education, Zhengzhou University of Science and Technology, Zhengzhou, China

**Keywords:** health belief, college students, sleep quality, physical activity, mobile phone addiction

## Abstract

**Background:**

Poor sleep quality has become a common health problem encountered by college students.

**Methods:**

Health belief scale (HBS), physical activity rating scale (PARS-3), mobile phone addiction tendency scale (MPATS) and Pittsburgh sleep quality index (PSQI) were adopted to analyze the data collected from survey questionnaires, which were filled out by 1,019 college students (including 429 males and 590 females) from five comprehensive colleges and universities from March 2022 to April 2022. The data collected from survey questionnaires were analyzed using SPSS and its macro-program PROCESS.

**Results:**

(1) Health belief, physical activity, mobile phone addiction and sleep quality are significantly associated with each other (*P* < 0.01); (2) physical activity plays a mediating role between health belief and sleep quality, and the mediating effects account for 14.77%; (3) mobile phone addiction can significantly moderate the effect size of health belief (*β* = 0.062, *p* < 0.05) and physical activity (*β* = 0.073, *P* < 0.05) on sleep quality, and significantly moderate the effect size of health belief on physical activity (*β* = −0.112, *p* < 0.001).

**Conclusion:**

The health belief of college students can significantly improve their sleep quality; college students’ health belief can not only improve their sleep quality directly, but also improve their sleep quality through physical activity; mobile phone addiction can significantly moderate the effect size of health belief on sleep quality, the effect size of health belief on physical activity, and the effect size of physical activity on sleep quality.

## 1. Introduction

Sleep quality is an important indicator of the quality of life. High-quality sleep can improve the physical and mental health of individuals (1, 2). Nowadays, sleep disorders have been defined as an increasingly serious public health concern (3), and college students are facing particularly acute sleep disorders. Data indicated that up to 60% of college students suffered from sleep problems such as insufficient sleep and difficulty in falling asleep (4), which not only affected their physical and mental health, but also affected their academic performance (5). Previous studies revealed that sleep quality is closely related to physical activity (6), mindfulness (7), health literacy (8), etc. With the popularity of the Internet and smart phones, mobile phone addiction has become a key factor that triggers sleep disorders (9). According to the emerging adulthood theory, college students in the stage of emerging adulthood may face multiple changes in cognition, emotion, role, and behavior (10), with a certain degree of self-identification and self-state ambiguity (11). This means that they may explore and establish a variety of lifestyles that align with their self-perception under changeable psychological states, and have greater likelihood of deviating from a healthy lifestyle (12), thus resulting in common problems faced by emerging adults such as low self-control, mobile phone addiction, weaker health belief, and lower physical activity levels (13). Without scientific and reasonable intervention or guidance, this may induce more serious health problems such as staying up late, insomnia, and alcohol abuse (14, 15). In view of this, college students were taken as research subjects of this study. The authors focused on the key variables that affect sleep quality, such as health belief, physical activity and mobile phone addiction, and thoroughly explored the influencing mechanism of health belief on college students’ sleep quality, in order to provide references for improving the sleep quality of college students.

### 1.1. Health belief and sleep quality

**Figure 1:**
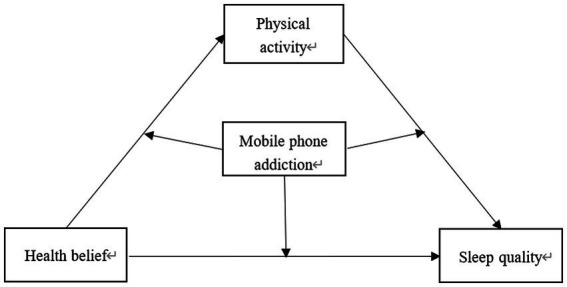
Hypothetical model diagram.

According to the health belief model (HBM), health belief is the psychological energy of human beings to perform health-related behaviors (16). The existing health knowledge and skills of individuals may not necessarily be converted into health-related behaviors, but health belief can deeply affect health-related behaviors (17). In the studies on sleep quality, health belief often serves as an important predictor variable (18). Studies also further revealed that individuals’ mental health can independently influence sleep quality, and positive state of mental health can exert a positive impact on sleep quality (19). Moreover, enhanced ideological and moral level of individuals can also create favorable conditions for the development of mental health (20) and the improvement of sleep quality (21). In addition, from the perspective of emerging adults, college students are prone to have sleep disorders due to the increase in negative psychological factors such as depression and anxiety (22), resulting in poor sleep quality. When they have a positive health belief, this may help them resist the interference of bad mood before sleep on the sleep process, and then improve the sleep quality (23). It is not difficult to find that health belief can affect sleep quality to varying degrees. Therefore, researchers should pay more attention to the relationship between health belief and sleep quality. Based on the theoretical and empirical studies mentioned above, this study proposed the Hypothesis 1 (*H*1): Health belief can significantly affect college students’ sleep quality (Figure 1).

### 1.2. The mediating role of physical activity

As an activity derived from human civilization, physical activity has been shown to have a positive impact on regulating individuals’ emotions and promoting their physical and mental health (24, 25). Previous studies have demonstrated a positive correlation between physical activity and active sleep quality indicators such as sleep efficiency (26), and revealed that adequate levels of physical activity are conducive to improving sleep quality (27). Additionally, Health Belief Model points out that health belief can effectively explain health-related behaviors such as physical activity (28, 29), and play a vital catalytic role in fostering exercise behavior intentions and exercise habits, thereby enhancing individuals’ enthusiasm for exercise (30). Under the drive of positive health belief, college students in the stage of emerging adulthood can strengthen their self-control ability by participating in physical activity regularly (31), effectively alleviate the unstable negative emotions that may arise during this period (32), help themselves shape a good psychological state (33, 34), and improve their sleep quality (35). In addition, according to the Self-Efficacy Theory (36), physical activity can be considered as a health-related behavior of individuals, which is driven by the strong health belief and can bring many health benefits to the body and mind, thus prolonging sleeping time, reducing sleep latency and improving sleeping effect and sleep quality (37). Therefore, physical activity may be an intermediary factor for individuals to perceive health belief, perform health-related behaviors and enjoy the benefits of good health. In this study, physical activity was taken as the mediator variable between health belief and sleep quality to better reveal the influencing mechanism of health belief on the sleep quality of college students. On this basis, Hypothesis 2 (*H*2) was put forward: Physical activity plays a mediating role between health belief and sleep disorder, that is, health belief may indirectly influence sleep quality through physical activity (Figure 1).

### 1.3. The mediating role of mobile phone addiction

Mobile phone addiction refers to the excessive addiction to various activities on mobile phones; it is a non-substance addiction or behavioral addiction that can impair the physiological, psychological and social functions of individuals (38). In fact, college students are at high risk of getting mobile phone addiction (39). Media Dependence Theory suggests that the greater an individual’s reliance on media (e.g., mobile phones) to satisfy their needs, the greater the impact of media on them. Previous studies revealed that smartphone addiction can trigger negative emotions such as depression, anxiety, stress, and loneliness among emerging adults (40), which can further lead to poor sleep quality (41–43). In the meanwhile, some studies further confirmed that mobile phone addiction can exert negative impact on positive psychological emotions such as learning input (44), psychological resilience (45), self-esteem and self-control (46), and negative impact on health belief and physical activity, which are considered as important health awareness and behavior. Based on the Flow Theory (47), smartphone addicts uncontrollably indulge in the pleasant experience brought by mobile phones. In this process, the content displayed on the mobile device screen may grab their attention and dominate their self-awareness, so that they tend to ignore other things around them and have lower self-control and willpower. For individuals, higher mobile phone addiction level is associated with immersive mobile phone user experience, vaguer self-awareness, weaker self-control and lower willpower, and vice versa. Therefore, it can be preliminarily concluded that mobile phone addiction exerts a moderating effect on individuals’ psychological intention and behavioral tendency. In this study, when college students are highly addicted to mobile phones, their positive psychological driving forces such as health belief may decrease dramatically, which in turn may reduce the impact of health belief on physical activity and sleep quality, and result in reduced physical activity behavior and poor sleep quality. Based on the theoretical and empirical studies mentioned above, this study proposed the Hypothesis 3 (*H*3): Mobile phone addiction can significantly moderate the effect size of health belief on sleep quality, the effect size of physical activity on sleep quality, and the effect size of health belief on physical activity (Figure 1).

## 2. Research design

### 2.1. Data and participants

In this study, random sampling method was adopted to select 1,250 students from five comprehensive colleges and universities to participate in the questionnaire survey from March 2022 to April 2022. After removing invalid questionnaires caused by insufficient time and insufficient answers, a total of 1,019 valid questionnaires were finally collected, with an effective rate of 81.25%.

Prior to the survey, the respondents were informed of the objective of the study and their voluntary participation in the questionnaire survey. It was emphasized that their data would be collected anonymously and kept confidential, and that the data collected would only be used for scientific research purposes and would not be disclosed. Finally, the data were collected after respondents signed the informed consent form.

### 2.2. Research tools

#### 2.2.1. Health belief scale

In this study, the health belief scale, which was specially developed for the Chinese population by Dan Su et al., was adopted (48). The scale includes four dimensions: virtue cultivation, healthy behavior habits, no symptoms of disease, and adaptive enjoyment, including a total of 28 items. The five-point Likert scale was adopted for scoring. Each item in the scale provides five possible answers ranging from “Strongly Disagree” to “Strongly Agree,” with 1–5 points, respectively. The scores of all items are added together. A higher score indicates a stronger health belief. The calculated *Cronbach’s α* coefficient of the health belief scale (HBS) was 0.954, indicating that the questionnaire has good reliability.

#### 2.2.2. Physical activity rating scale

The physical activity rating scale compiled by Deqing Liang was adopted to rate physical activity level (49). The scale was mainly used to evaluate physical activity levels from three aspects: intensity, frequency and duration of each physical activity, and to define the high, medium and low levels of physical activity. Score of physical activity level = physical activity intensity × (physical activity duration—1) × physical activity frequency. The 5-point Likert scale was adopted for scoring, with scores ranging from 1 to 5 points for each item. The total score is 100 points. If the total score ≤19 points, it is at low physical activity level; if the total score is between 20 and 42 points, it is at medium physical activity level; and if the total score ≥43 points, it is at high physical activity level. The calculated *Cronbach’s α* coefficient of the physical activity rating scale was 0.709, indicating that the scale has good reliability.

#### 2.2.3. Mobile phone addiction tendency scale

In this study, the mobile phone addiction tendency scale (MPATS) compiled by Jie Xiong et al. was adopted (50). The scale mainly covers four aspects: withdrawal symptoms, highlighted behavior, social comfort, mood alteration, including a total of 16 items. The 5-point Likert scale was adopted for scoring. Each item in the scale provides five possible answers ranging from “Strongly Disagree” to “Strongly Agree,” with 1–5 points, respectively. The scores of all items are added together. Higher score indicates higher mobile phone addition level. The calculated *Cronbach’s α* coefficient of the Mobile Phone Addiction Tendency Scale (MPATS) was 0.924, indicating that the scale has good reliability.

#### 2.2.4. Pittsburgh sleep quality index

In this study, the Pittsburgh sleep quality index (PSQI), developed by Byusse et al. was adopted (51). The scale includes 18 items, which are divided into seven factors, including sleep quality, sleep latency, sleep duration, sleep efficiency, sleep disorder and daytime dysfunction. A 4-point scoring system was adopted, with each item scored from 0 to 3 based on the degree of sleep quality. The total score is obtained by summing up the scores of all items, with a higher score indicating poorer sleep quality. The *Cronbach*’s α coefficient of the questionnaire was calculated to be 0.876, indicating good reliability of the scale.

### 2.3. Research procedure

Firstly, after obtaining informed consent from both the schools and the students themselves, trained teachers and students were designated as the primary examiners to conduct the questionnaire survey at the class level. During the survey, standardized instructions were provided to inform respondents about the content and requirements of the questionnaire. Secondly, the collected data were statistically analyzed using SPSS 26.0, and moderated mediation analysis was conducted using the SPSS macro-program PROCESS.

## 3. Results and analysis

### 3.1. Common method deviation test

Harman’s single factor test was used to examine potential common method bias. All questions related to the four variables (health belief, physical activity, mobile phone addiction and sleep quality) were included in the exploratory factor analysis. 12 factors with eigenvalues greater than 1 were calculated, which explained 69.784% of total variance. The first factor explained 17.880% of the variance, which was lower than the critical value of 40%. These findings indicate that there was no obvious common method bias in the investigation process.

### 3.2. Demographic characteristics

Table 1 shows the analysis of 1,019 respondents, comprising 429 males (42.10%) and 590 females (57.90%). The age distribution included 156 students aged 18 or below (15.31%), 353 students aged 19 (34.64%), 306 students aged 20 (30.03%), 141 students aged 21 (13.84%), and 63 students aged 22 or above (6.18%). In terms of academic year, 452 were freshmen (44.36%), 492 were sophomores (48.28%), and 75 were juniors (7.36%). The participants were from different fields of study: 495 were from liberal arts programs (48.58%), 311 were from science and engineering programs (30.52%), and 213 were from the physical education program (20.90%).

**Table 1:** Description of demographic characteristics.

		Number of respondents (%)	Health belief		Physical activity		Mobile phone addiction		Sleep quality	
			*M* ± *SD*	*T*/*F* value	*M* ± *SD*	*T*/*F* value	*M* ± *SD*	*T*/*F* value	*M* ± *SD*	*T*/*F* value
Gender	Male	429 (42.10%)	112.1 ± 20.13	3.6***	27.16 ± 24.321	11.9***	38.63 ± 12.278	3.488***	6.59 ± 2.384	1.165
	Female	590 (57.90%)	107.84 ± 16.351		11.78 ± 13.131		41.13 ± 9.815		6.42 ± 1.936	
Age	18 or below	156 (15.31%)	112.08 ± 19.201	3.061*	16.41 ± 18.969	1.006	39.52 ± 12.329	1.106	6.14 ± 2.024	5.124**
	19	353 (34.64%)	109.97 ± 17.838		18.59 ± 20.763		40.07 ± 10.497		6.33 ± 1.823	
	20	306 (30.03%)	108.01 ± 17.23		18.56 ± 19.24		41 ± 10.908		6.48 ± 2.167	
	21	141 (13.84%)	107.39 ± 19.067		17.16 ± 20.596		38.82 ± 10.969		7.06 ± 2.592	
	22 or above	63(6.18%)	114.54 ± 18.32		21.97 ± 22.737		39.92 ± 10.47		7.02 ± 2.466	
Grade	Freshman	452(44.36%)	109.83 ± 18.105	1.207	18.31 ± 20.651	5.931**	39.37 ± 11.332	1.694	6.22 ± 1.947	10.635***
	Sophomore	492(48.28%)	109.02 ± 17.986		17.08 ± 18.74		40.65 ± 10.49		6.61 ± 2.16	
	Junior	75(7.36%)	112.44 ± 19.433		25.64 ± 24.376		40.6 ± 11.889		7.35 ± 2.729	
Specialty	Liberal arts majors	495(48.58%)	107.18 ± 16.115	19.515***	12.04 ± 13.644	196.563***	40.84 ± 10.588	2.767	6.46 ± 1.921	7.65**
	Science and engineering majors	311(30.52%)	109 ± 17.765		14.01 ± 14.969		39.73 ± 10.25		6.22 ± 2.092	
	PE majors	213(20.90%)	116.24 ± 21.377		38.89 ± 25.395		38.82 ± 12.702		6.95 ± 2.568	

*P < 0.05, **P < 0.01, ***P < 0.001.

Significant differences were found in the scores of health belief, physical activity, and mobile phone addiction among college students of different genders (*p* < 0.001). College students of different ages also showed significant differences in the scores of health belief and sleep quality (*p* < 0.05), while those of different grades had significant differences in the scores of physical activity and sleep quality (*p* < 0.01). In addition, college students of different majors exhibited significant differences in the scores of health belief, physical activity, and sleep quality (*p* < 0.01).

### 3.3. The mean, standard deviation and correlations of different variables

Descriptive statistics and correlation analysis results in Table 2 indicated that health belief, physical activity, mobile phone addiction and sleep quality have significant correlation with each other (*p* < 0.01). Significant positive correlations were found between health belief and physical activity (*r* = 0.293, *p* < 0.01), and between mobile phone addiction and sleep quality (*r* = 0.242, *p* < 0.01). On the other hand, health belief showed significant negative correlations with mobile phone addiction (*r* = −0.280, *p* < 0.01) and sleep quality (*r* = −0.219, *p* < 0.01), while physical activity showed significant negative correlations with mobile phone addiction (*r* = −0.255, *p* < 0.01) and sleep quality (*r* = −0.169, *p* < 0.01). Taken together, the correlations between variables reached a significant level, suggesting that health belief and physical activity can improve sleep quality, while mobile phone addiction can lead to sleep disorders. Further statistical analysis can be conducted.

**Table 2:** The standard deviation, mean and correlation matrix of each variable.

Variables	Health belief	Physical activity	Mobile phone addiction	Sleep quality
Health belief	1			
Physical activity	0.293**	1		
Mobile phone addiction	−0.280**	−0.255**	1	
Sleep quality	−0.219**	−0.169**	0.242**	1

*P < 0.05, **P < 0.01, ***P < 0.001.

### 3.4. Mediating effect analysis

The PROCESS plug-in compiled by Hayes was adopted, and Model 4 was selected to test the mediating effect among the relationship of health belief, physical activity, and sleep quality under controlled conditions (including gender, age, grade and major). The results indicated that (see Table 3), in the absence of mediator variables, health belief had a significant negative impact on sleep quality (*β* = −0.202, *t* = −6.510, *p* < 0.001), indicating that improving health belief can enhance sleep quality. Therefore *H*1 is supported. Health belief had a significant positive impact on physical activity (*β* = 0.196, *t* = 7.206, *p* < 0.001), while physical activity had a significant negative impact on sleep quality (*β* = −0.153, *t* = −4.287, *p* < 0.001). These findings suggest that health belief can enhance physical activity, which in turn improves sleep quality. These findings preliminarily support the mediating role of physical activity in the relationship between health belief and sleep quality among college students. In addition, Bootstrap analysis was used to conduct the mediation path test. The results showed that (see Table 4), in terms of direct effects, the lower limit of the 95% confidence interval of health belief on sleep quality was −0.2338, and the upper limit was −0.1101. This interval did not contain 0, indicating that health belief can exert negative impact on sleep quality directly. The effect value was −0.172, and direct effects accounted for 85.23%. For the mediating effect of physical activity, the lower limit of the 95% confidence interval was −0.0475, and the upper limit was −0.0154. This interval did not contain 0, indicating that physical activity played a mediating role between health belief and sleep quality. The effect value was −0.0298, and mediating effects accounted for 14.77%. The establishment of the mediating effect indicates that health belief can not only directly influence the sleep quality of college students, but also indirectly affect their sleep quality through physical activity. Therefore, it can be inferred that *H*2 is supported.

**Table 3:** Mediating effect analysis of physical activity.

Variables	Sleep quality				Physical activity				Sleep quality			
	*β*	*t*	*P*	95%CI	*β*	*t*	*P*	95%CI	*β*	*t*	*P*	95%CI
Gender	−0.004	−0.127	0.899	[−0.073, 0.064]	−0.193	−6.273	0	[−0.253,-0.132]	−0.034	−0.954	0.34	[−0.234,-0.110]
Age	0.071	1.819	0.069	[−0.006,0.148]	−0.006	−0.171	0.865	[−0.073,0.062]	0.07	1.812	0.07	[−0.222,-0.083]
Grade	0.075	1.919	0.055	[−0.002,0.151]	−0.013	−0.386	0.7	[−0.080,0.054]	0.073	1.883	0.06	[−0.103,0.036]
Major	0.087	2.468	0.014	[0.018,0.156]	0.335	10.862	0	[0.275,0.396]	0.138	3.74	0	[−0.006,0.147]
Health belief	−0.202	−6.51	0	[−0.263,-0.141]	0.196	7.206	0	[0.142,0.0.249]	−0.172	−5.457	0	[−0.003,0.149]
Physical activity									−0.153	−4.287	0	[0.066,0.211]
*R*	0.25				0.531				0.281			
*R* ^2^	0.062				0.282				0.079			
*F*	13.461				79.445				14.473			

All variables in the model are standardized data. This is also applicable to the table below.

**Table 4:** Bootstrap analysis of the significance test results of mediating effect.

Influence path	Effect value	Boot	Boot CI	Boot CI	Relative effect ratio/%
		Standard deviation	Lower limit	Upper limit	
Health belief → Sleep quality	−0.1720	0.0315	−0.2338	−0.1101	85.23%
Health belief → Physical activity → Sleep quality	−0.0298	0.0082	−0.0475	−0.0154	14.77%
Total effect	−0.2018	0.0310	−0.2627	−0.1410	100%

### 3.5. Moderated mediation model test

**Figure 2:**
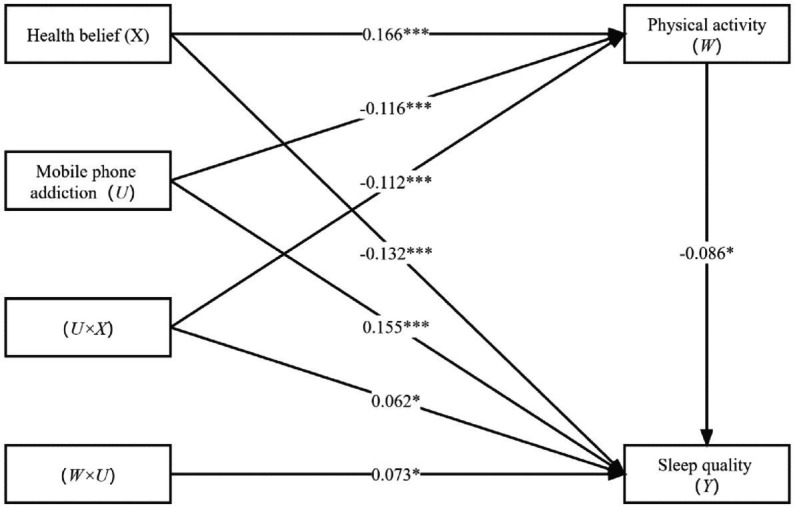
Moderated mediation model.

In terms of moderating effects (Table 5 and Figure 2), the PROCESS plug-in was used and Model 59 was selected to test the moderated mediation model under controlled conditions (including gender, age, grade and major). After incorporating mobile phone addiction as a variable into the mediation model, the interaction between health belief and mobile phone addiction exerted a significantly negative impact on physical activity (*β* = −0.112, *t* = −4.491, *P* < 0.001), and a significantly positive impact on sleep quality (*β* = 0.062, *t* = 2.096, *P*< 0.05); the interaction between physical activity and mobile phone addiction exerted a significantly positive impact on sleep quality (*β* = 0.073, *t* = 2.446, *p* < 0.05). Therefore, the direct influence path of health belief on sleep quality, the first half path and the second half path of the mediating effect of health belief on sleep quality through physical activity were significantly influenced by mobile phone addiction, confirming the validation of *H*3.

**Table 5:** Moderated mediation model test.

Variables	Physical activity				Sleep quality			
	*β*	*t*	*P*	95%CI	*β*	*t*	*P*	95%CI
Gender	−0.176	−5.859	0	[−0.235,--0.117]	−0.04	−1.137	0.256	[−0.108,0.029]
Age	−0.017	−0.508	0.612	[−0.083,0.049]	0.078	2.034	0.042	[0.003,0.153]
Grade	0.003	0.088	0.93	[−0.063,0.068]	0.058	1.535	0.125	[−0.016,0.133]
Major	0.33	10.95	0	[0.271,0.389]	0.125	3.451	0.001	[0.054,0.196]
Health belief	0.166	6.073	0	[0.112,0.220]	−0.132	−4.105	0	[−0.195,-0.069]
Mobile phone addiction	−0.116	−4.055	0	[−0.172,-0.060]	0.155	4.736	0	[0.094,0.220]
Health belief × mobile phone addiction	−0.112	−4.491	0	[−0.161,-0.063]	0.062	2.096	0.036	[0.004,0.120]
Physical activity					−0.086	−2.345	0.019	[−0.157,-0.014]
Physical activity × mobile phone addiction					0.073	2.446	0.015	[0.014,0.132]
*R*	0.564				0.345			
*R* ^2^	0.318				0.119			
*F*	67.396				15.186			

**Figure 3:**
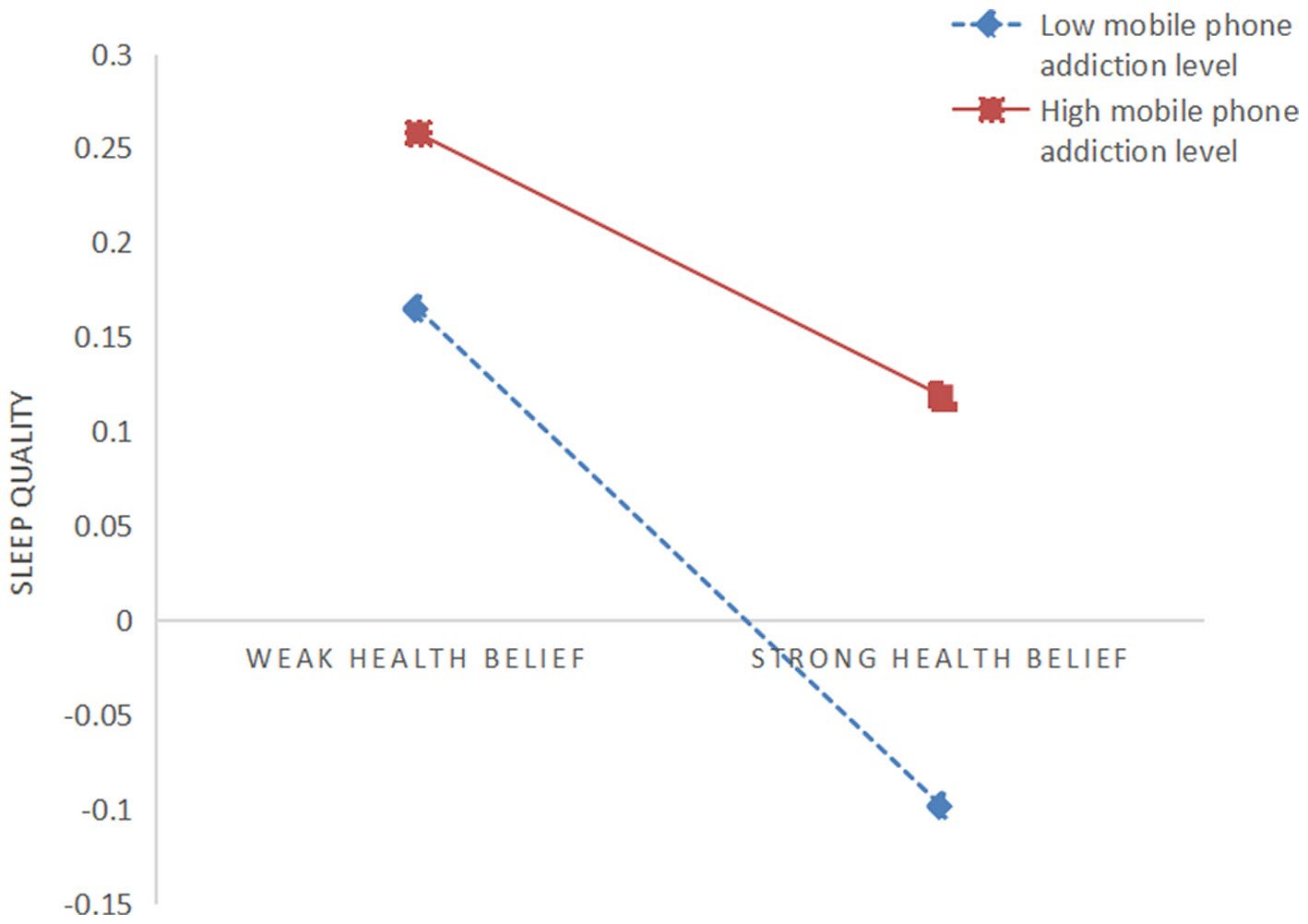
The mediating effect of mobile phone addiction on the relationship between health belief and sleep quality.

Simple slope analysis further revealed that, in terms of direct effects (as shown in Figure 3), students with low scores of mobile phone addiction (*M*-1SD) experienced a significantly negative impact of health belief on sleep quality (*simple slope* = −0.194, *t* = −4.1764, *P* < 0.0001). In contrast, students with high scores of mobile phone addiction (*M* + 1SD) did not show a significantly negative impact of health belief on sleep quality (*simple slope* = −0.070, *t* = −1.709, *p* > 0.05). These findings suggest that mobile phone addiction can significantly moderate the effect size of health belief on sleep quality. Specifically, as mobile phone addiction scores increase, the negative impact of health belief on sleep quality gradually decreases (i.e., sleep quality declines). Conversely, as mobile phone addiction scores decrease, the negative impact of health belief on sleep quality gradually becomes more prominent (i.e., sleep quality improves).

**Figure 4:**
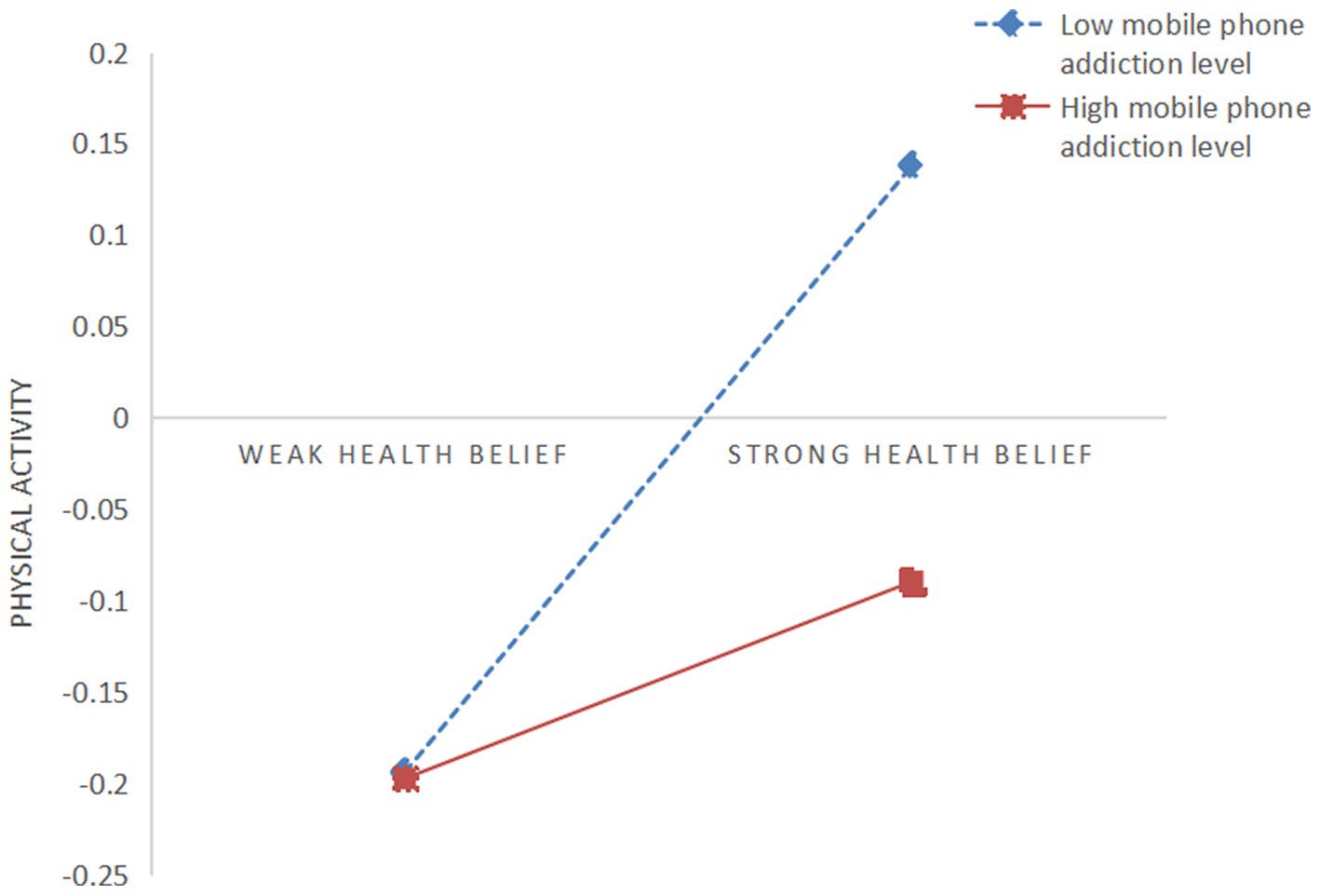
The mediating effect of mobile phone addiction on the relationship between health belief and physical activity.

In the first half of mediating effect (as shown in Figure 4), students with low scores of mobile phone addiction (*M*-1SD) experienced a significantly positive impact of health belief on physical activity (*simple slope* = 0.278, *t* = 7.264, *p* < 0.01); however, students with high scores of mobile phone addiction (*M* + 1SD) did not show a significantly positive impact of health belief on physical activity (*simple slope* = 0.0538, *t* = 1.508, *p* > 0.05). These findings suggest that mobile phone addiction can significantly moderate the effect size of health belief on physical activity. Specifically, as mobile phone addiction scores increase, the positive impact of health belief on physical activity gradually decreases; as mobile phone addiction scores decrease, the positive impact of health belief on physical activity gradually becomes more prominent.

**Figure 5:**
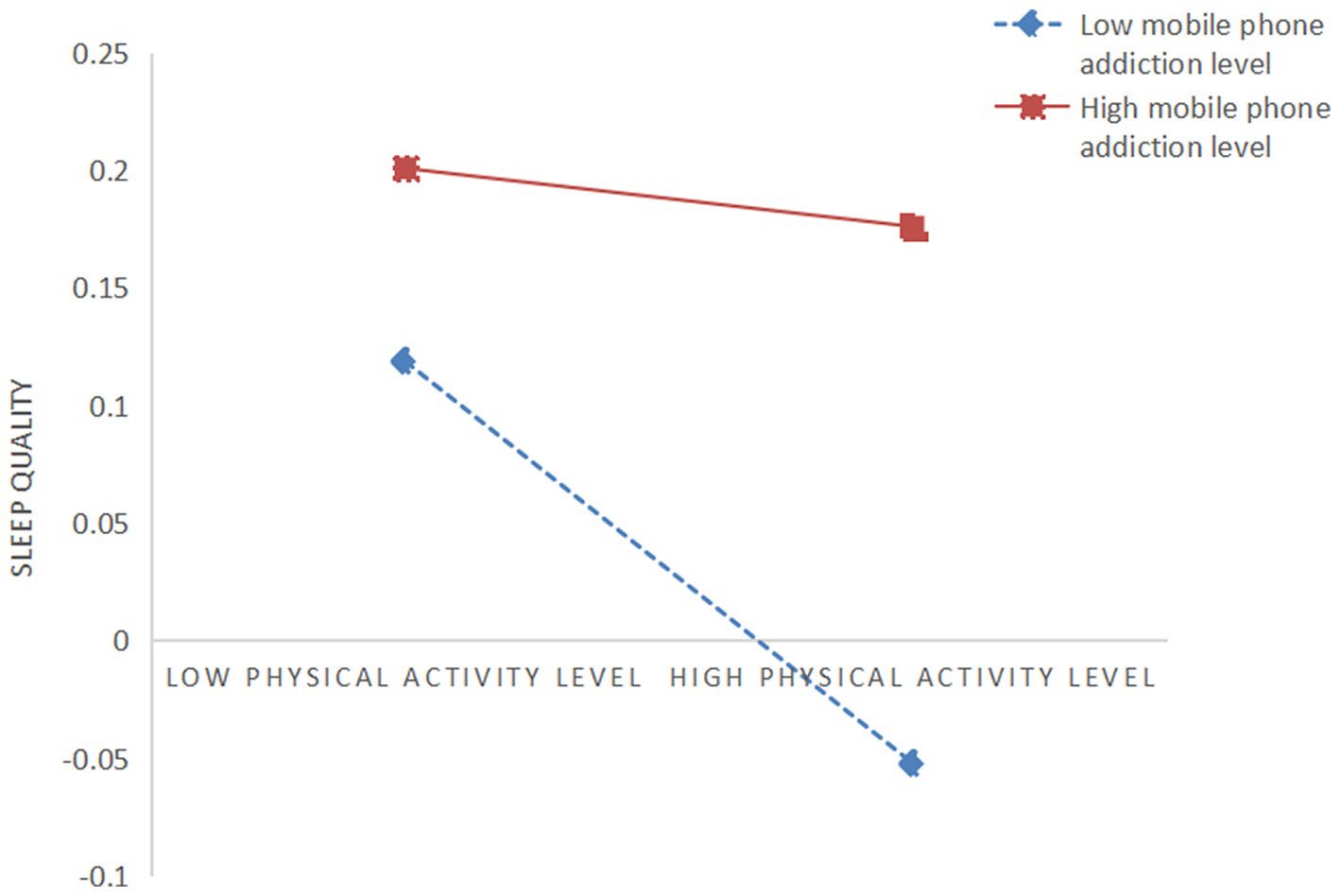
The moderating effect of mobile phone addiction on the relationship between physical activity and sleep quality.

In the second half of mediating effect (as shown in Figure 5), students with low scores of mobile phone addiction (*M*-1SD) experienced a significantly negative impact of physical activity on sleep quality (*simple slope* = −0.159, *t* = −3.735, *P*<0.001); however, students with high scores of mobile phone addiction (*M* + 1SD) did not show a significantly negative impact of physical activity on sleep quality (*simple slope* = −0.012, *t* = −0.242, *p* > 0.05). These findings suggest that mobile phone addiction can significantly moderate the effect size of physical activity on sleep quality. Specifically, as mobile phone addiction scores increase, the negative impact of physical activity on sleep quality gradually declines (sleep quality declines); as mobile phone addiction scores decrease, the negative impact of physical activity on sleep quality gradually becomes more prominent (i.e., sleep quality improves).

## 4. Discussion

### 4.1. Health belief and sleep quality

This study revealed that health belief can exert significantly negative impact on sleep quality. In other words, for college students, higher score of health belief is associated with lower score of sleep quality and better sleep quality. The findings support the views of previous relevant studies (52). According to the Health Belief Model and Implicit Theory, if individuals have a more positive health belief, they generally think that their health status is more changeable (53), and they are more willing to take actions to improve sleep quality, which is conducive to improving sleep status (54). Specifically, those college students who have a strong health belief have a clearer understanding of the value of health literacy and healthy lifestyle, fully stimulate their subjective initiative, effectively improve their self-efficacy for health-related behaviors, and generate positive health cognition and behavioral tendency, thereby promoting the establishment of a stable, long-term healthy lifestyle in emerging adulthood and facilitating healthy physiological and psychological development (54). In the meanwhile, this mechanism may also contribute to the balanced secretion of individual brain neurons in the long run, which can stabilize psychological emotions and improve health literacy and sleep quality (53). To sum up, when college students have a positive health belief, they are more likely to regulate their own mental health status, and improve their sleep quality by optimizing their own health cognition and behaviors.

### 4.2. The moderating role of physical activity

This study revealed that physical activity exerted a mediating effect between health belief and sleep quality. This means that health belief can indirectly affect college students’ sleep quality through physical activity. On the one hand, health belief can exert significantly positive affect on the physical activity behaviors of college students. This is more consistent with the previous research findings that “people with a strong health belief have a higher intention to do physical activity” (55). On the other hand, physical activity behavior can exert significantly negative impact on the sleep quality of college students. This means that students with higher physical activity level and lower sleep quality score tend to have better sleep quality. This is consistent with previous research results (56). According to the Self-Efficacy Theory and the analysis based on the health belief model, it can be considered that health belief is the internal drive to promote health-related behaviors, which can guide individuals to form health-related behavior intentions and establish stable and mature health ideologies and behavioral habits in emerging adulthood (28). Therefore, college students with strong health belief may have relatively strong health efficacy, such as health cognition, health intention and health behavior, and are more likely to actively eliminate the constraints that restrict their participation in physical activities under the help, support, advice or reminder of others (57), and improve the possibility of their own health-related behaviors such as physical activity (36), in order to provide feedback about the feeling of health efficacy generated by health belief. Furthermore, college students with strong health belief tend to be more sensitive to their own physical health, and can perceive the susceptibility and vulnerability to diseases. As a result, they can provide a stable precursor thinking and result prediction for their own participation in physical activity, effectively improve their health awareness, fully stimulate behavior decision-making and participation motives, and promote their own participation in leisure exercise. In addition, physical activity is an active health behavior driven by the health belief of college students (58, 59). With increasing participation intensity, it can effectively alleviate negative emotions such as stress, anxiety, and depression brought about by emerging adulthood (32), ultimately leading to an improvement in sleep quality. From the perspective of physiological mechanism, physical activity can improve the vagus nerve function, adjust cortisol level (60, 61), and stimulate the pineal gland to secrete melatonin (62), while mobilizing the human hypothalamus to control the body’s heat dissipation mechanism, triggering and increasing slow-wave sleep, and improving sleep quality (63). Meanwhile, proper physical activity can induce a state of relative fatigue in the body and stimulate the body’s recovery mechanisms, which can shorten the time it takes for individuals to fall asleep, promote deeper sleep, and ultimately improve overall sleep quality (64). Generally speaking, the mediating role of physical activity can be interpreted as follows: Driven by the health belief that college students actively seek to improve their sleep quality, a health behavior that can bridge the gap between the two is generated, and this health behavior can effectively implement the driving instructions of the health belief, ultimately improving the sleep quality of college students through physical practice.

### 4.3. The moderating effect of mobile phone addiction

This study also revealed that mobile phone addiction can significantly moderate the effect size of health belief and physical activity on sleep quality, as well as the effect size of health belief on physical activity. Specifically, as mobile phone addiction scores increase, the positive impact of health belief on physical activity gradually decreases, and the negative impact of health belief and physical activity on sleep quality also decreases, i.e., college students’ sleep quality shows a decline; as mobile phone addiction scores decrease, the positive impact of health belief on physical activity gradually becomes more prominent, and the negative impact of health belief and physical activity on sleep quality also gradually becomes more prominent, i.e., college students’ sleep quality improves. The results are consistent with those of previous studies (65), and have further verified the basic view of Flow Theory (47). On the one hand, according to the analysis based on Flow Theory, when being immersed in the mobile phone world, those college students with higher mobile phone addiction level are more likely to uncontrollably indulge in pleasant experience brought by mobile phones. During this process, human body may constantly accelerate the activities of endocrine system, gradually release adrenaline, catecholamine, dopamine and other exciting hormones, which can make human body stay in a state of excitement, trigger circadian rhythm disorder and other factors that can directly affect sleep quality (66, 67), and thus lead to poor sleep quality. In the meanwhile, being immersed in the virtual world provided by mobile phones can lead to a gradual decline in individuals’ self-awareness and time consciousness, resulting in a psychological state where they forget to maintain or improve their physical and mental health. In the long run, this not only reduce the level their health belief and the physical activity behaviors driven by their health belief, but also diminishes the positive effect of physical activity in linking health belief and sleep quality. Eventually, this may lead to the problem of reduced sleep quality due to insufficient health awareness and lack of physical activity. On the other hand, the emerging adulthood theory suggests that individuals in the stage of emerging adulthood may experience a certain level of self-awareness ambiguity (11). Moreover, college students have just completed their college entrance examinations and entered a relatively relaxed university environment, where the constraints of parents, teachers, and school systems continue to decrease. This may make them more susceptible to developing an excessive dependence on mobile phones. When they use their phones for extended periods of time, they may suffer from rumination psychology, such as regret, remorse and the desire to compensate, which can drive them to develop more severe negative psychological emotions such as anxiety, panic, stress, and depression (68, 69). This can lead to a range of physical and mental problems, including social anxiety and a lack of interest in physical activity, eventually resulting in physiological and psychological sleep disorders, loss of health belief, and various problems that hinder sleep (70, 71). Taken together, it can be concluded that mobile phone addiction not only leads individuals to indulge in the physical and mental excitement of the virtual world created by mobile phones, causing them to neglect their health beliefs and health behaviors, but also results in negative rumination and reduces their health beliefs and health behaviors after excessive use of mobile phones, thus ultimately affecting their sleep quality.

### 4.4. Research significance and limitations

By building a moderated mediation model to explore the relationship between health belief, physical activity, mobile phone addiction and sleep quality, this study further verified the relationship between health belief and sleep quality and its influencing mechanism. Therefore, this study can provide some inspiration for improving the sleep quality of college students in China. Firstly, colleges and universities should strengthen the cultivation of health belief among college students through various means (such as strengthening the concept of health education, improving the health education system, reforming the health education system, introducing health education teaching staff, enriching the content of health education, strengthening health education publicity, building a health evaluation system and a health education feedback mechanism, etc.), comprehensively enhance the health belief level of college students in a multi-field and precise manner, and thus lay a solid foundation for improving the sleep quality of college students. Secondly, colleges and universities should pay attention to the systematization of physical education teaching. By systematizing physical education curriculum training programs, physical education knowledge structure and sport project skills, we can ensure that students will be able to master proficient sport skills and promote physical and mental health through proficient sport skills in the process of doing exercise, so as to provide auxiliary force for improving the sleep quality of college students. Finally, colleges and universities can popularize the negative impact of mobile phone addiction through education and publicity, actively advocate the healthy concept of rational use of mobile phones, improve college students’ awareness on the hazards of mobile phone addiction, and guide college students to relieve their dependence on mobile phones from the perspective of individuals’ subjective consciousness. In the meanwhile, colleges and universities should take the initiative to create a positive campus interpersonal atmosphere and exercise atmosphere; design diversified interpersonal interaction activities and sports activities to shift students’ attention from mobile phones to the communication between teachers and students and the communication between students, so as to alleviate and avoid mobile phone addition and reduce the interfering factors that affect the sleep quality of college students.

Admittedly, although this study preliminarily revealed the influencing mechanism of health belief, physical activity, mobile phone addiction and sleep quality using a moderated mediation model, there are still some deficiencies that need to be further improved. First of all, the research data was mainly collected through self-assessment questionnaires, which may have certain methodological effects (such as memory bias and conceptual understanding). In future research, questionnaires should be collected through multiple channels (such as teachers, parents, and classmates). Secondly, this study adopted a cross-sectional study design, which can only be used to explore the relationship between health belief, physical activity, mobile phone addiction and sleep quality, and cannot make causal inference. In future research, experimental research or longitudinal design should be adopted. Finally, in addition to the variables mentioned in this study, there must be other relevant factors that can affect the sleep quality of college students. Future research should make further exploration and reveal the influencing mechanism of sleep quality among college students more comprehensively.

## 5. Conclusion

(1) Health belief can exert significantly negative impact on sleep quality. In other words, health belief can improve college students’ sleep quality. (2) Physical activity plays a mediating role between health belief and sleep quality, that is, health belief can improve college students’ sleep quality by improving physical activity level. (3) Mobile phone addiction can significantly moderate the effect size of health belief on sleep quality, the effect size of physical activity on sleep quality, and the effect size of health belief on physical activity. When the mobile phone addiction level decreases, the negative impact of health belief and physical activity on sleep quality becomes more prominent, and the positive impact of health belief on physical activity becomes more prominent, thereby improving the sleep quality of college students. When the mobile phone addiction level increases, the negative impact of health belief and physical activity on sleep quality decreases, and the positive impact of health belief on physical activity also decreases, leading to poorer sleep quality among college students.

## Data availability statement

The raw data supporting the conclusions of this article will be made available by the authors, without undue reservation.

## Ethics statement

The studies involving human participants were reviewed and approved by Biomedical Research Ethics Subcommittee of Henan University Henan University. Written informed consent to participate in this study was provided by the participants’ legal guardian/next of kin.

## Author contributions

XG proposed topics, wrote, and revised the paper. CL collected and analyzed data, wrote, and revised the paper. BH analyzed data, gave instructions, and revised the paper. PX collected data and revised the paper. CQ collected data and revised the paper. All authors contributed to the article and approved the submitted version.

## Funding

This study is a planned project of Liaoning Sport Science Society (Grant No. 2022LTXH086).

## Conflict of interest

The authors declare that the research was conducted in the absence of any commercial or financial relationships that could be construed as a potential conflict of interest.

## Publisher’s note

All claims expressed in this article are solely those of the authors and do not necessarily represent those of their affiliated organizations, or those of the publisher, the editors and the reviewers. Any product that may be evaluated in this article, or claim that may be made by its manufacturer, is not guaranteed or endorsed by the publisher.
